# Internet Usage by Patients with Multiple Sclerosis: Implications to Participatory Medicine and Personalized Healthcare

**DOI:** 10.1155/2010/640749

**Published:** 2010-06-30

**Authors:** Izabella Lejbkowicz, Tamar Paperna, Nili Stein, Sara Dishon, Ariel Miller

**Affiliations:** ^1^Rappaport Faculty of Medicine and Research Institute, Technion - Israel Institute of Technology, P.O. Box 9649, Haifa 31096, Israel; ^2^Department of Community Medicine and Epidemiology, Carmel Medical Center, Haifa 34362, Israel; ^3^Multiple Sclerosis Center, Carmel Medical Center, Haifa 34362, Israel

## Abstract

Online health information and services for patients were suggested to improve symptom management and treatment adherence, thereby contributing to healthcare optimization. This paper aimed to characterize multiple sclerosis (MS) patients Internet usage. Information regarding browsing habits, Internet reliability, and the medical team's attitude to information collected online was obtained by questionnaires from MS patients. Data was compared between nonbrowsers, browsers on MS topics, and browsers on non-MS topics only. From the 96 patients recruited, 61 (63.5%) performed MS-related searches. The most viewed topics were “understanding the disease” and “treatments”. Patients reported that the information helped coping with MS and assured them of the appropriateness of their therapy. Shorter disease duration was correlated with higher Internet activity. Disabled patients were more interested in online interaction with specialists and support groups. This paper suggests that MS patients benefit from online information, and it emphasizes the importance of resources tailored to patients needs.

## 1. Introduction

Research in the last decades has shown that the more involved patients have better health outcomes, due to their increased treatment adherence and awareness to preventive tests. In addition, the availability of information has changed patients' expectations regarding the extent to which they should be involved in decisions on their own care [1, 2]. Medicine is becoming *participatory*: patients are increasingly engaged and active participators in personal choices about illness and well-being [3].

One of the major sources of health information in recent years is the Internet. In the US, 75% of Internet users (about 60% of the total population) search about health information online [4, 5]. Nevertheless, there is a gap between the number of patients actually searching for information and those reporting what they found to their physicians. Therefore some patients are exposed to information that is not reliable, and of which their physicians are not aware nor able to provide their reaction, or opinion, [6, 7]. 

To date, only few studies have assessed the issue of Internet usage by patients; Atreja et al. reported that patients usually look for information before and after medical visits and use the Internet to understand medical terms [8]. 

In another study, Hay et al. [9] reported that the majority of MS participants searched for information on the Internet prior to the first visit to an MS clinic. Internet activity was correlated with income but not with education, marital status, health status, or gender. Although information found on the Internet did not replace the information obtained from the physician, two-thirds of the patients were reluctant to discuss Internet information with their physician. The authors suggested that patients were concerned that online searches might be perceived as a lack of confidence in the physician skills. The fact that patients are unlikely to discuss search results with physicians may have implications for patient adherence to treatment [9].

The quality and information content of websites available for the MS patient was found to be variable, with a few sites offering nearly all of the information needs of people with MS [10]. A few websites, as the one from the “MS Centers of Excellence” [11], go beyond information and support self-monitoring of the disease. Since MS patients experience a vast array of symptoms that may exacerbate or decrease over time and may suffer also from memory impairment, detailed registration of the symptoms can improve disease management, by allowing more accurate reports to the physician. A similar system was developed by Lowe-Strong and McCullagh who built a computerized visual interface for self-recording of pain associated with MS [12]. 

MS patients expressed interest in a web-based portal that would include self-monitoring of MS symptoms, prescriptions orders, laboratory results retrieval, online patient education, updates on MS research, and, most importantly, timely communication with the medical team [8, 13]. 

MS prevalence in Israel is in the range of the medium to high zone [14], with over 3000 patients currently registered in the Israeli National MS Register. Although the Internet is frequently used in Israel by an estimated 56% to 70% of the population [15, 16], to the best of our knowledge there are no reports on the use of the Internet for seeking health information by patients, and specifically by MS patients. 

The objectives of this paper were (1) to assess the percentage of Internet users and the percentage of seekers of information on MS among the patients of our clinic, (2) to characterize the topics on MS most searched in the Internet and the outcomes of these searches, (3) to assess the patients' attitudes towards Internet information, and (4) to assess the impact of disease duration and disability on information seeking. 

## 2. Materials and Methods

### 2.1. Recruitment Procedure

MS patients who visited the MS clinic at Carmel Medical Center, a major referral MS center in Northern Israel, during the spring of 2009, who were 18 years and older and able to communicate in Hebrew, were invited to participate in the study. Patients willing to participate completed questionnaires about Internet reliability and accessibility, their browsing habits, demographic data, and their opinion about the medical team's attitude to the information they collected through the Internet. Clinical data was obtained from participants' medical records. 

The study received ethics approval from Carmel Medical Center's Helsinki Committee, and all participants received explanations on the study objectives and signed informed consents. 

### 2.2. Statistical Analysis

Statistical analysis was performed using SPSS 15.0 software. The continuous variables are presented as means, S.D. and medians, and the categorical variables are presented as percentages. Comparisons between groups of patients were done by using One Way ANOVA followed by independent *T*-test for the continuous variables, and by Kruskal-Wallis followed by Mann Whitney for the ordinal variables. Chi square test or Chi square exact test were used as appropriate for the categorical variables. *P* < .05 was considered statistically significant.

## 3. Results

### 3.1. Demographic and Clinical Data of Study Participants

A total of 103 patients agreed to participate and 96 (93%) completed the questionnaire. As in other studies based on self-filled questionnaires, response rate was not the same for all questions, and therefore percentage of respondents was calculated separately for each question.

The average age of patients was 43.2 years and female/male ratio was 2.4, similar to the ratio reported in other studies [17]. Sixty-two percent of the participants (56 out of 90) had more than 12 years of education which is lower than that reported in studies of MS patients in other countries [13, 18–21]. Mean disease duration since diagnosis was 7.7 years, and mean EDSS (Expanded Disability Status Scale) was 2.6, indicating a mild disability level for this patient cohort. Although in this paper only patients fluent in Hebrew were recruited, participants included a variety of ethnicity groups and countries of origin, characteristic of the Israeli population. 

Sixty-one patients (63%) used the Internet and searched for topics related to MS (MS Internet users- MSIU). Eighteen patients (19%) used the Internet for general issues not related to MS (general Internet users- GIU) and 17 patients (18%) did not use the Internet (noninternet users—NIU). 

Comparison of demographic and clinical data between the 3 groups of patients can be seen in Table 1. The main differences between NIU and MSIU were the older age and longer disease duration of the NIU group.

Among NIU, the principal reasons stated for not using the Internet to search about MS were lack of computer operating skills (6 out of 17 patients, 35%), lack of knowledge on computer search tools (4 out of 17 patients, 23%), or lack of access to a computer (4 out of 17 patients, 23 %). Among GIU the principal reasons were lack of interest in information about MS (8 out of 18 patients, 44%) and lack of knowledge on search tools (4 out of 18 patients, 22%).

### 3.2. Additional Sources of Information about MS

The physician and the nurse were the principal source of information on MS for patients in all 3 groups: 94% of NIU (15 out of 16 respondents), 87% of GIU (13 out of 15 respondents) and 94% of MSIU (47 out of 50 respondents). Other sources of information reported were leaflets, newspapers, and the television. MSIU also searched more significantly for information on newspapers than GIU (3 out of 15 respondents, 20%; compared to 27 out of 49 respondents, 55%; *P* = .02) and in the television (4 out of 14 respondents, 29%; compared to 22 out of 47 respondents, 47%; *P* = .03). The most common source of information on the disease for NIU was leaflets (7 out of 11 respondents, 64%).

### 3.3. Browsing Habits of Internet Users

Browsing habits of GIU and MSIU were similar. Over 80% of participants in both groups browsed at least two times a week and browsed in Hebrew sites. Other languages used were English, Russian and Arabic. 

The most frequent uses of the Internet were for Email (16 out of 18 GIU patients, 89%, and 54 out of 59 MSIU patients, 91%), work-related issues and tourism, and entertainment. Regarding the usage of online health services (not related to MS), although the majority of patients in both groups reported the usage of the Internet for setting medical appointments and retrieving lab results, the groups differed in their use of search services: MSIU used the Internet more frequently to search for physicians (32 of 47 MSIU patients, 68%, compared to 4 of 12 GIU patients, 33%, *P* = .04) and to search for medicines, diseases, and medical tests (47 of 52 MSIU patients, 90%, compared to 6 of 13 GIU patients, 46%, *P* = .001).

### 3.4. MS Topics Searched and Outcomes of Searches

**Figure 1:**
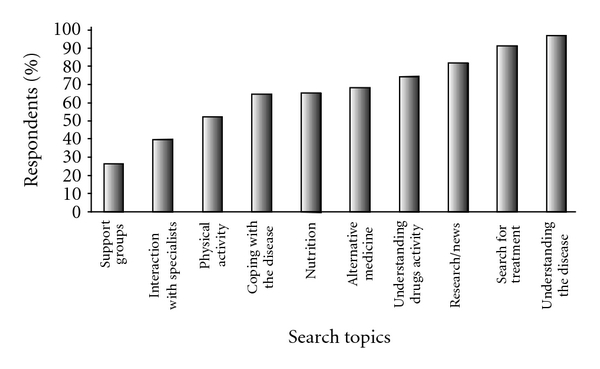
Topics of search about MS (response rate: 80% to 92%).

There was high variability in the percentage of MSIU patients interested in different information topics (Figure 1). Over 90% of the patients searched for information in order to understand the disease and to find a treatment. The lower rates were for interactive topics such as support groups/contact with other patients and interaction with specialists. 

**Figure 2:**
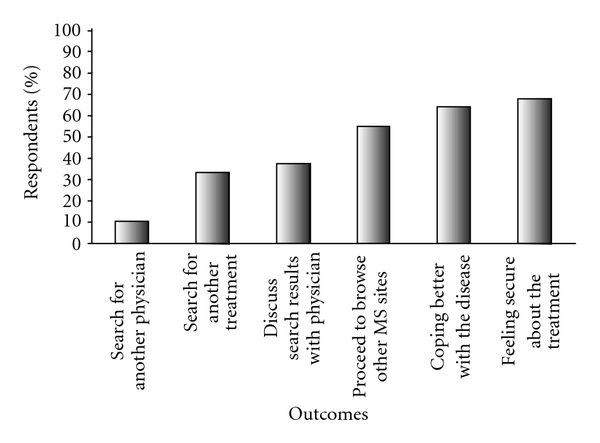
Outcomes of searches about MS (response rate: 77% to 87%).

The principal outcomes following searches in the Internet were patient confidence in the treatment received and better coping with the disease (Figure 2).

Similarly to what was reported in other studies [6, 7, 9], only one-third of the patients discussed the information they gathered on the Internet with their physician.

The websites most visited for searches related to MS were patients' associations sites (73%—36 of the 49 respondents) and academic sites (69%—36 of the 52 respondents). Commercial medical sites were visited by 26 out of 51 respondents (51%), pharmaceutical companies' sites were visited by 20 out of 43 respondents (46%), *health maintenance organizations* (HMOs) sites were visited by 17 out of 47 respondents (36%), and hospital sites were visited by 11 out of 43 respondents (26%).

### 3.5. Patients' Attitudes to Information, the Internet, and the Medical Team's Approach to Information Gathered on the Internet

GIU and MSIU attitudes towards information, the Internet, and the medical team's attitude to information gathered on the Internet were compared. Participants were asked on a 4 point scale if they do not agree at all (1) or completely agree (4) to each one of the attitudes' related statements. Responses 1 and 2 were grouped into “do not agree” and responses 3 and 4 were grouped into “agree”. The most interesting results are shown in Table 2. The results indicate that patients rely on online information and are satisfied with the information available to them. Most patients stated that information about MS contributed to their ability to cope with the disease, especially among MSIU, and only about one third of patients in both groups stated that information about the disease frightens them. 

Although participants did not feel encouraged by the medical team to search for information, as reported also in other studies [22], about half felt the medical team was happy to discuss with them information they found and the majority reported feeling part of the decision the making process.

### 3.6. Relation between Information Needs and Disease Duration and Disability

In order to assess if the information needs of MSIU change according to disease duration, we compared disease durations for patients that searched for information on each of the topics presented in Table 1 and those that did not include that topic in their searches. No statistically significant difference in disease duration was found in any of the search topics. However, two of the attitude statements studied (presented in Table 2) appeared to be influenced by the disease duration of the patients: the disease duration of MSIUs who stated that they increase their searches for information when the disease worsens was longer than that of patients that did not state so (mean 7.8 years compared to 4.3 years, *P* = .02), and the disease duration of MSIUs who stated that they feel part of the treatment decision making was significantly longer than that of patients who did not state so (mean 7.2 years compared to 4.5 years; *P* = .04). 

For assessing if the information needs of patients are related to disease disability, patients were grouped according to their EDSS score in 3 groups: lower or equal to 3, between 3 and 5.5, and equal to or higher than 5.5. We tested for each of the topics searched if there is a difference between the EDSS scores of patients who searched information on that topic versus patients that did not include the topic in their searches. Patients with EDSS 5.5 and higher were significantly more interested in interacting with specialists than patients with 3 < EDSS < 5.5 (4 of the 6 respondents, 67% compared to 1 of the 10 respondents, 10%; *P* = .04). High EDSS score patients were also more interested in support groups via the Internet, than patients with 3 < EDSS < 5.5 and patients with EDSS ≤ 3 (4 of the 6 respondents, 67%; 2 of the 9 respondents, 22%; and 7 of the 34 respondents, 21%, resp., *P* = .06). 

**Figure 3:**
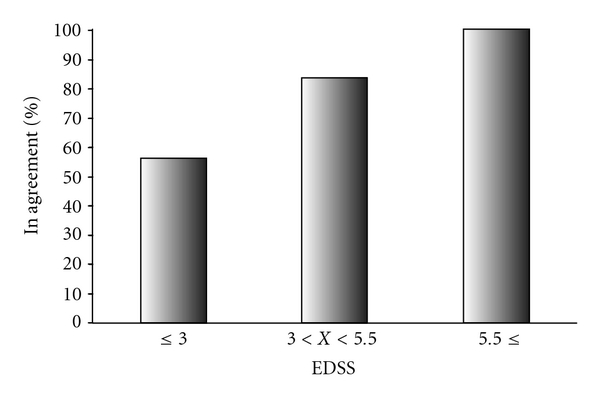
Association between patients disability and interest in Internet information on coping strategies of other patients. Patient disability groups are defined by EDSS range (*x*-axis). The percentage of respondents that agreed with the sentence: “I would be happy to read about how other patients cope with MS” is indicated by % of in agreement. (*P* for Trend = .01, response rate >95%)

Regarding patients' attitudes to information, patients who were more disabled appeared to be more interested in reading about other patients' coping strategies with MS. All 6 patients with EDSS 5.5 and higher expressed interest on this topic, whereas the patients in the lower EDSS groups displayed a trend of decreasing interest with the lower disability levels (10 patients of 12 respondents with EDSS 3 < EDSS < 5.5, 83% and 23 out of 42 respondents with EDSS ≤ 3, 56%, *P* for trend = .01; Figure 3).

## 4. Discussion

The main findings of this study were that the majority of MS patients browsed the Internet for information on MS, and their information needs were correlated to disease duration and severity. Participants considered online information as accessible and reliable, and claimed it helped them cope with MS and raised confidence in their therapy program. 

### 4.1. Internet Usage by MS Patients

Most MS patients from our clinic that browse the Internet search for information related to the disease (MSIU group), similarly to findings from previous studies on MS [9, 23] and other chronic diseases [24]. Nevertheless, the medical team remains their principal source of information. Among the noninternet users (NIUs), the reason for not using the Internet was mainly lack of computer skills, similarly to findings of a previous study of cancer patients [25], which is in line with their older average age compared to MSIU patients who searched for MS topics. Interestingly, although the GIU patients had Internet skills that were similar to the MSIU patients, yet they did not search for information on MS, mostly due to “lack of interest”. Although the average age of this group was similar to that of the NIU group, since their Internet skills resembled that of the MSIU group, their age difference may indicate an age-dependent attitude biased towards the physician-dependent paternalistic healthcare approach. A study with a larger number of GIU is necessary to better understand the reasons for their Internet behavior. In any case, it should be clarified that appropriate information on the disease and the treatments given by physicians remains the main goal to reach.

### 4.2. Reliability and Accessibility of Information on the Internet

The majority of patients considered information on MS available on the Internet to be as reliable as in books and more accessible. The reliability and credibility of health information on the Internet and its effect on healthcare have been frequently discussed. There is a general assumption that low-quality information on the Internet may lead to potential harm, although no evidence can be found in the literature [26]. Studies that measure the impact of information on healthcare objectively, for example, by measuring health status of informed compared to less informed patients, are difficult to perform, due to the presence of confounding factors such as participants' interest in online information and health status of participants before the intervention [27]. Due to the limited number of participants in this study, a consequence of the interval time stipulated for recruitment, the effect of confounding factors like disease type and severity on change of health status could not be assessed and the evaluation of the contribution of online information to healthcare was based on the patients' self-impression of benefit. No harmful effects of the information on the Internet were reported, rather, the information was perceived to have positive effects. The first one was the ability to cope with the disease, which has already been connected to how informed the patient is. Lode et al. [28] reported on a study on MS-related coping styles that optimizing the information process, especially in the early phase of the disease, may induce coping styles that produce a better adaption to living with MS.

Another positive effect of information seeking was confidence in the treatment received, which can improve treatment adherence. Lately, the term “adherence” has replaced the term “compliance” with respect to treatment, in order to emphasize the importance of the patients' independent role in treatment decision-making process [29]. Nonadherence is seen as an unnecessary risk for further morbidity and mortality, as well as a waste of health care resources, and is a significant issue in MS management [30]. Information is an important key to patient empowerment in decision making and optimization of therapy, contributing to increased adherence. This is especially true when multiple treatment options are present, as in MS. 

Most participants did not discuss the online information with their physicians, similar to findings of other studies on MS [9], rheumatologic diseases [24], and cancer [25] The physician therefore may not be aware of unreliable information the patients may have been exposed to, which may have affected their coping behaviors, and accordingly, disease management. 

### 4.3. Association between Disease Duration and Disability and Information Needs of MS patients

Our finding that patients with shorter disease duration tended to search more for information on the Internet reinforces studies that have emphasized the importance of supplying information to recently diagnosed MS patients [28, 31]. On the other hand, patients with longer disease duration searched more for information when the disease worsens, an indication of the change of information needs over time. They also felt more part of the decision making in their treatment, which may be due to a longer interaction over the years between the patient and the medical team. 

Patients with higher disability expressed preference to interaction through the Internet with specialists and support groups, and were more interested in reading about coping approaches of other patients with the disease. This may indicate that the Internet serves a social role for these patients, whose disability limit their accessibility to social contacts, as well as to medical support systems. These results emphasize, as suggested also in previous studies [32, 33], that information should be tailored to suit patients according to their needs, and that the accessibility to online information is of exceptional importance to the disabled. 

### 4.4. Development and Implementation of Websites

The development of quality websites to a patient's population requires the knowledge of the specific conditions related to their disease. Furthermore, data on browsing habits including frequency, languages, and use of online services can allow development of sites adequate to these habits. Another issue to be taken into account is the MS patient's limitations in accessing the Internet, due to disease-related impairments such as visual disturbances, fatigue, and cognitive and memory problems in MS [8]. Physicians then can take advantage of the availability of reliable sites by referring patients to appropriate information and discussing the information as part of the health management plan. 

The results presented here can contribute to the development and implementation of websites dedicated to MS patients.This paper outlines the information topics of most interest for MS patients and the association between information needs and disease stage. MS clinics may improve medical care by providing relevant information which is patient-centered and online as possible. Additionally, MS patient societies, HMOs, and pharmaceutical companies, may play an important role as information sources by maintaining informative and updated websites, catered to patient usage. The Internet could also be used for patients education, for example, by providing tutorials on how to inject medications and potential adverse events, and could serve as a tool for data collection in MS research, through the use of Internet-based surveys [34]. 

When developing Internet resources for MS, it should be taken into account that some patients do not use the Internet due to lack of the necessary skills. These patients should be identified by the medical team and should either receive the necessary support to be able to use this important tool or should be provided with the relevant information through other means.

## 5. Conclusions

The results of this study, which to the best of our knowledge is the first to present an association between MS patients' clinical and demographic characteristics and their attitudes and browsing behaviors with respect to disease-related information on the Internet, indicate that Internet usage is well accepted by MS patients and contributes to patients' well being. Although Internet based information is not often discussed with the treating physician, extending the patient-physician interaction to the web is likely to increase patient accessibility to reliable information, and contribute to their health management. Internet usage may contribute to improved and personalized healthcare by offering information that suits the patient's specific needs and perceptions regarding the stage of his/her disease, impairment, socio economic and computer literacy level, and ethnicity viewpoints. Additional studies, with larger number of participants and objective assessment of information effect on health status, will contribute to development of useful patient-oriented sites that will aid in positive coping strategies. Tailored information will lead to more empowered patients towards the goals of modern medicine: to be both personalized and participatory. 

##  Conflict of Interest

None of the authors declared conflict of interest.

## Figures and Tables

**Table 1:** Demographic and clinical characteristics of study participants.

		NIU	GIU	MSIU	*P*-value
		Non Internet Users	General Internet Users	MS-Internet Users	
*N* (%)		17 (18)	18 (19)	61 (63)	
Age: Mean ±SD		47.7 ± 8.2	46.9 ± 8.8	40.9 ± 10.4	.01^a^
Female/male (ratio)		16/1 (16.0)	11/7 (1.6)	41/20 (2.0)	.67

EDSS^b^ *N* (%)	≤3	8 (47)	12 (67)	43 (70)	.47
	>3 & <5.5	7 (41)	4 (22)	12 (20)	
	≥5.5	2 (12)	2 (11)	6 (10)	

Disease Duration years ± SD^c^		9.6 ± 5.3	9.6 ± 5.1	6.6 ± 5.1	.02^c^

Education *N* (%)^d^	≤12 years	10 (59)	3 (17)	21 (38)	.04^d^
	>12 years	7 (41)	15 (83)	34 (62)	

I have a computer at home *N*(%)^e^		10 (71)	18 (100)	59 (98)	.002^e^

Marital Status *N* (%)	Married	14 (82)	13 (72)	45 (74)	.73
	Single	2 (12)	2 (11)	11(18)	
	Divorced	1 (6)	3 (17)	5 (8)	

Ethnic origin *N* (%)	Jews	14 (82)	17 (94)	53 (87)	.50
	Arabs	3 (18)	1 (6)	8 (13)	

Country of birth *N* (%)	Israel	8 (47)	11 (61)	49 (80)	
	Past USSR	3 (18)	4 (22)	4 (7)	.04
	Others	6 (35)	3 (17)	8 (13)	

^a^ *P* = .02 between NIU & MSIU, *P* = .02 between GIU & MSIU.

^b^EDSS = Expanded Disability Status Scale.

^c^ *P* = .04 between NIU & MSIU, *P* = .03 between GIU & MSIU.

^d^ *P* = .01 between NIU & GIU.

^e^ *P* = .004 between NIU & MSIU, *P* = .03 between NIU & GIU.

**Table 2:** Participants' attitudes about information and the internet: number and percentage of patients that agree with attitude statements.

Attitude statement	GIU-*N* (%) General Internet Users	MSIU-*N* (%) MS-Internet Users	*P*
It is easier to find information on MS in the Internet than in books	13 (93)	53 (88)	>.99
It is easier to ask the physician than to search the Internet	15 (94)	33 (56)	.005
Information in books is more reliable than information in the Internet	5 (38)	18 (33)	.75
People with other diseases find more information than people with MS	2 (25)	12 (21)	>.99
Information about MS helps me cope with the disease	7 (47)	46 (78)	.02
Information about MS frightens me	5 (31)	22 (37)	.69
I search for more information when the disease worsens	6 (40)	40 (69)	.04
I would be happy to read about how other patients cope with MS	5 (33)	39 (66)	.04
I would be happy to make contact with my medical team through the Internet	6 (43)	41 (68)	.07
I feel part of the decision making in my treatment	9 (69)	48 (84)	.24
The medical team encourages me to look for information about MS	1 (8)	14 (25)	.27
The medical team is happy to discuss with me new information I found	6 (50)	30 (59)	.75
